# Supplementary material for: The lncRNA landscape of breast cancer reveals a role for DSCAM-AS1 in breast cancer progression
Source: Nat Commun. 2016 Sep 26;7:12791. doi: 10.1038/ncomms12791 (PMC5052669; doi:10.1038/ncomms12791)
Supplement: Supplementary Information — Supplementary Figures 1-8. [file ncomms12791-s1.pdf]

Supplementary Figure 1

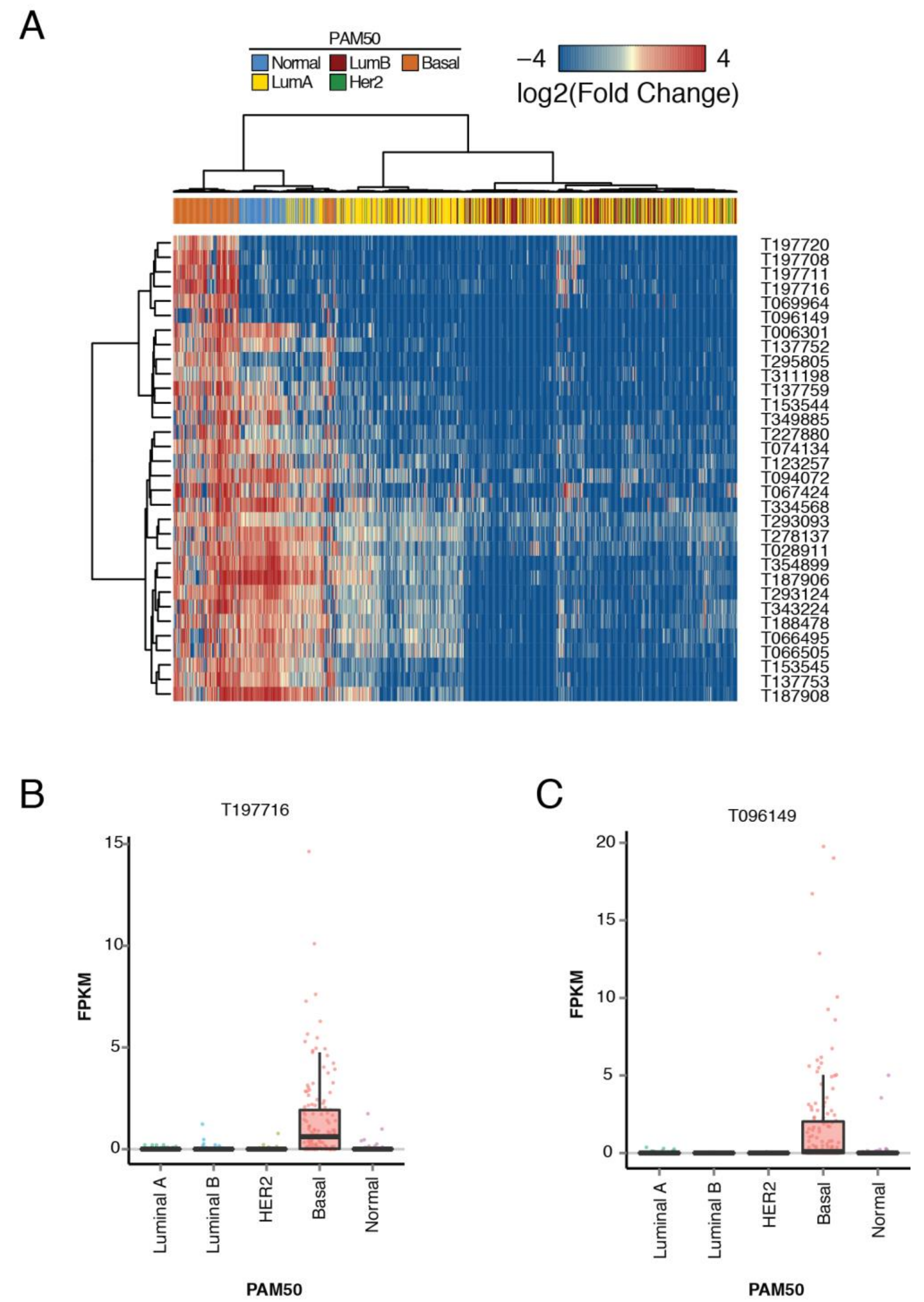

Supplementary Figure 1. Discovery of basal specific lncRNAs. (**a**) heatmap depiction of basal specific lncRNAs delineated in **Figure 1b**. 32 lncRNAs were identified as being basal specific. Expression values are depicted as log2 of the fold-change over the median of the ER-negative samples. Unsupervised hierarchical clustering was done on both lncRNAs and patients (n=946). PAM50 classification is shown

above heatmap. (**b**) Representative example of expression pattern for MiTranscriptome transcript T197716 across the PAM50 subtypes. (**c**) Representative example of expression pattern for MiTranscriptome transcript T096149 across the PAM50 subtypes.

Supplementary Figure 2

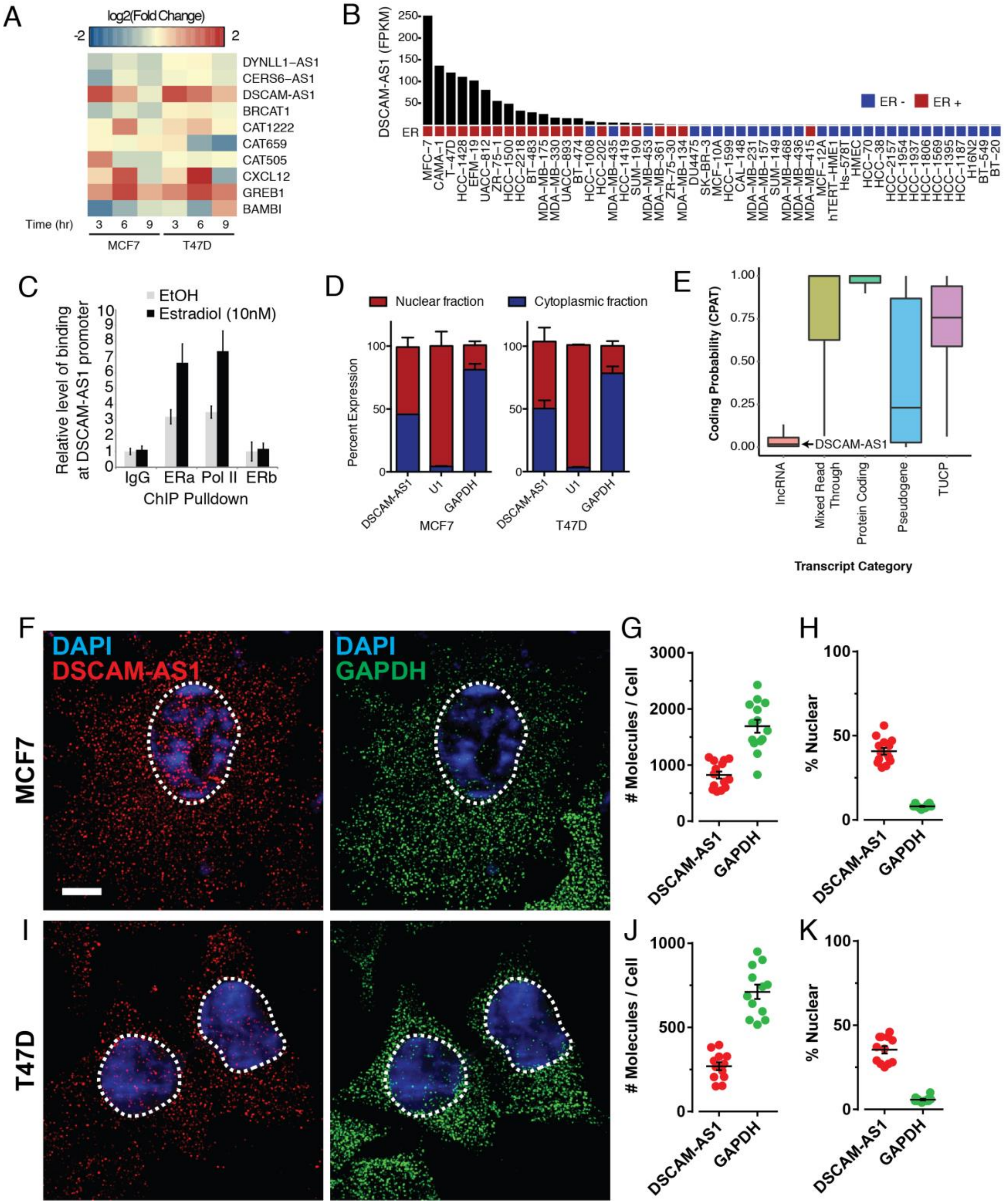

Supplementary Figure 2. Characterization of DSCAM-AS1. (**a**) Heatmap representation of qPCR validation of estrogen responsiveness in MCF7 and T47D cell lines. Heatmap values are the log2 of the fold change over time zero for each cell line. The most highly expressed cancer and ER-positive associated lncRNAs were tested. *GREB1* and *CXCL12* were used as a positive control. And *BAMBI* used as a negative control. (**b**) Expression of *DSCAM-AS1* in 50 breast cancer cell lines from RNA-seq data. ER status of cell line shown in blue/red below expression bar. (**c**) qPCR of the DSCAM-AS1 promoter following ChIP for ERa, ERb, and RNA Pol II following 12hr estradiol or DMSO vehicle stimulation. Expression normalized to IgG pulldown. Error bars represent the s.e.m. for three biological replicates. (**d**) Cellular localization of *DSCAM-AS1* in MCF7 and T47D cells. qPCR performed following cellular fractionation. U1 and GAPDH were used as controls for the nuclear and cytoplasmic fractionation, respectively. Error bars represent the s.e.m. of three biological replicates. (**e**) Boxplot depiction of the CPAT coding potential scores for all MiTranscriptome transcripts by transcript category. CPAT coding potential score for *DSCAM-AS1* (4 isoforms with CPAT score ranging from 0.015-0.016) is highlighted. (**f**) Representative pseudocolored image of an MCF7 cell probed for *DSCAM-AS1* (red, left) and *GAPDH* (green, right). Nucleus is stained with DAPI (blue). White dotted line represents nuclear boundary. Scale bar, 10 µm. B and C, Scatter plots representing the number of DSCAM-AS1 or GAPDH molecules per MCF7 cell. (**g**) and the percentage of transcripts that localize in the nucleus (**h**). Black line and error bars depict the mean and s.e.m. respectively (n =14 cells). (**i**) Representative pseudocolored image of a T47D cell, probed and stained as in (**f**). (**j** and **k**) Scatter plots are similar to (**g**) and (**h**) respectively (n =12 cells).

Supplementary Figure 3

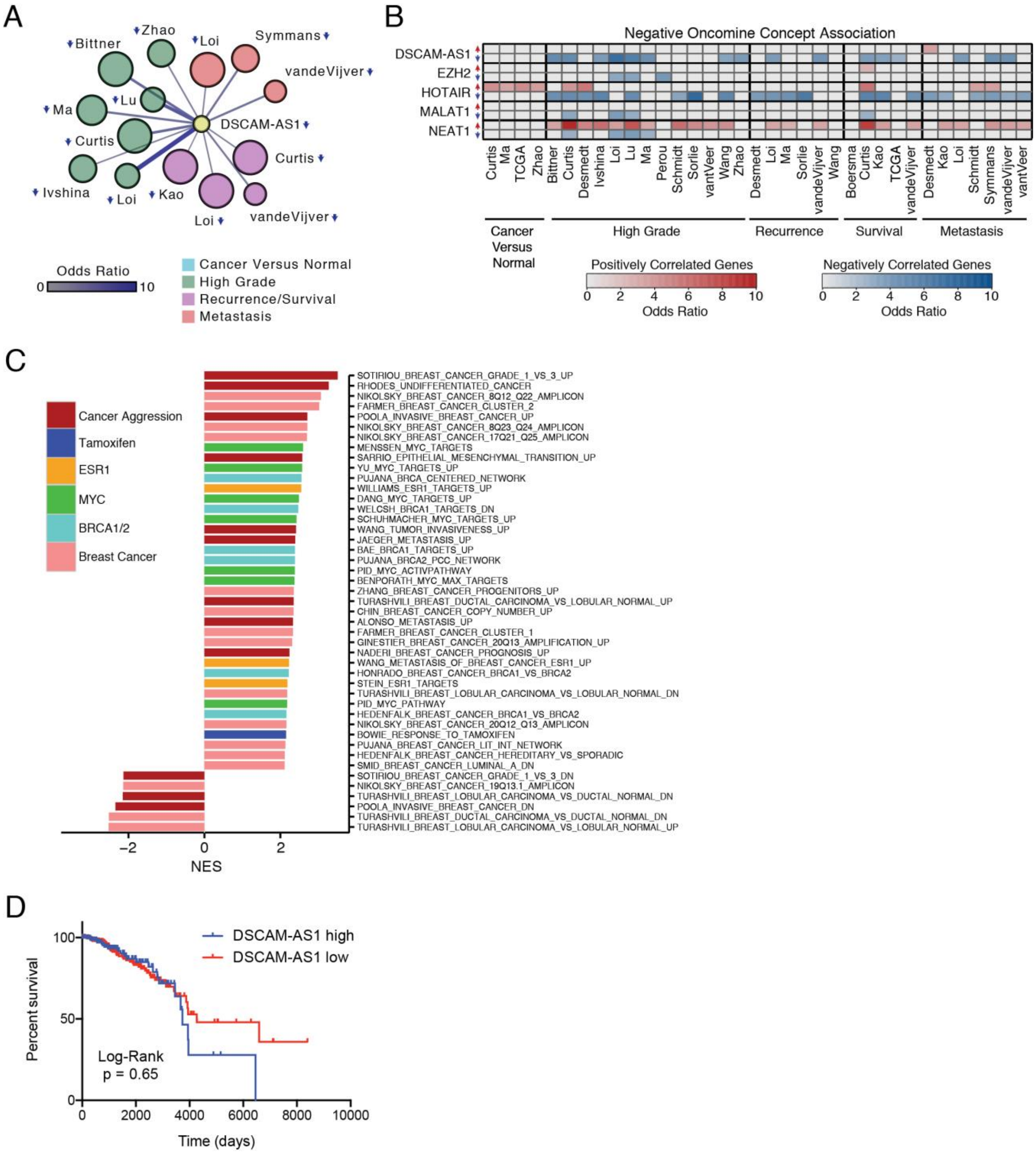

Supplementary Figure 3. *DSCAM-AS1* clinical associations. (**a**) Cytoscape depiction of the overlap between the 150 genes most negatively correlated with *DSCAM-AS1* and clinical signatures from Oncomine[35] for breast cancer clinical outcomes (i.e., recurrence, survival, and metastasis), high cancer

grade, and cancer versus normal. All significant associations with an odds ratio > 4 are shown (Fisher's p-value < 1e-4). Size of node reflects the size of the gene signature, and the thickness/redness of the line represents the magnitude of the odds ratio. (**b**) Heatmap displaying the overlap between the top 150 genes correlated to *DSCAM-AS1*, *EZH2*, *HOTAIR*, *MALAT1*, and *NEAT1* and the genes negatively associated with various breast cancer Oncomine clinical signatures for cancer versus normal, high clinical grade, recurrence, survival, and metastasis. For each gene, the top row depicts the odds ratio for the positively correlated genes (red), and the bottom row represents the odds ratio for the negatively correlated genes (blue). The first name of the author for each clinical study is listed. (**c**) Preranked GSEA performed for the genes correlated to *DSCAM-AS1* using the ER-positive breast cancer samples. NES values for all significant (GSEA FDR < 1e-5) MSigDB[75] signatures related to cancer aggression and breast cancer are plotted. (**d**) Kaplan-Meier analysis of survival in the TCGA breast cohort for ER-positive samples. Samples were divided based on DSCAM-AS1 expression (> 10 FPKM = DSCAM-AS1 high; < 1 FPKM = DSCAM-AS1 low). Statistical significance determined by Log-Rank test.

Supplementary Figure 4

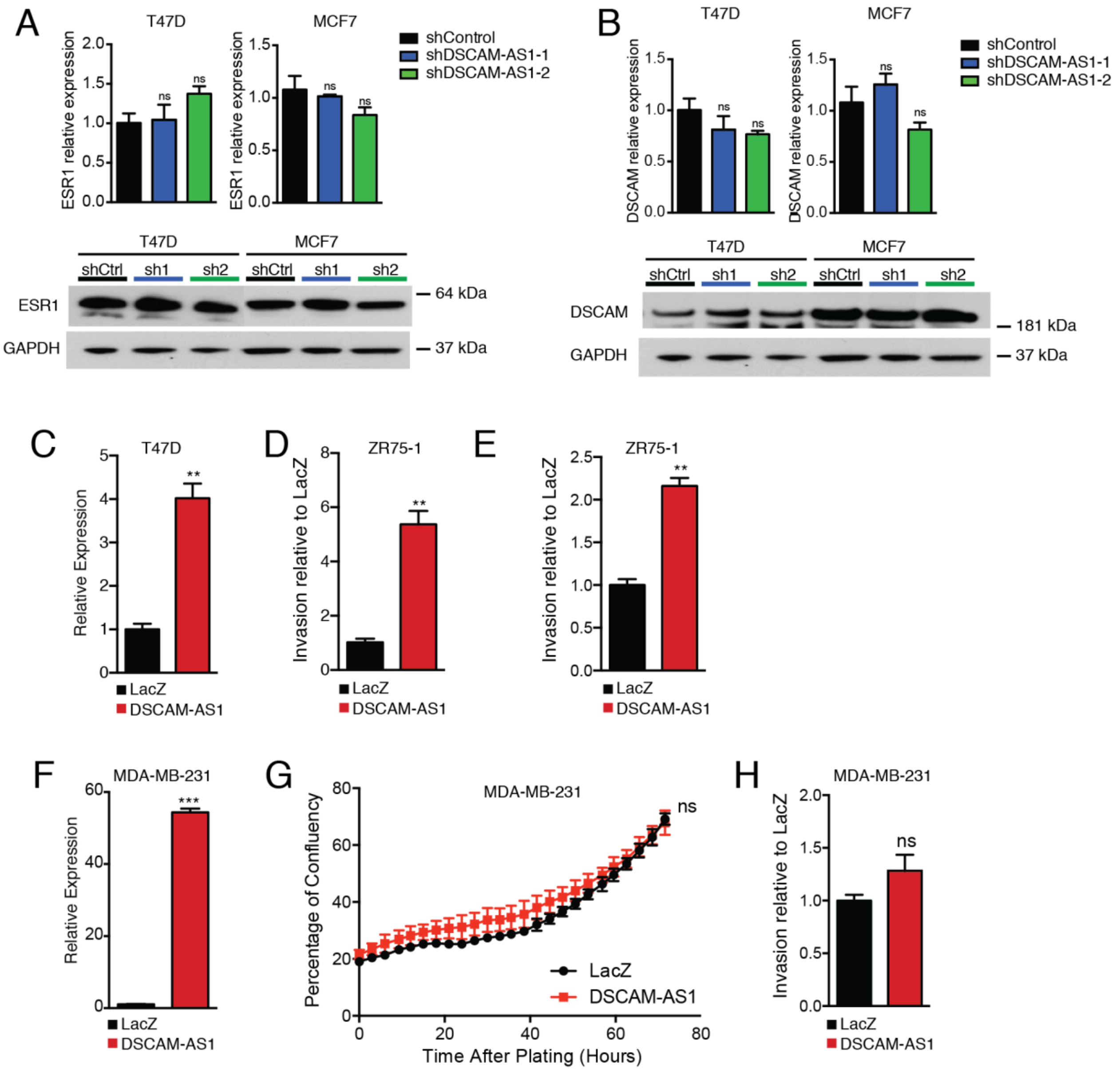

Supplementary Figure 4. *DSCAM-AS1* knockdown and overexpression mediate cancer phenotype. (**a**) RNA (top) and protein (bottom) levels of *ESR1* following shRNA knockdown of *DSCAM-AS1* in MCF7 and T47D cells. qPCR expression normalized to shControl. Error bars represent the s.e.m. for three biological replicates. Blot is representative of three independent experiments. (**b**) RNA (top) and protein (bottom) levels of *DSCAM* following shRNA knockdown of *DSCAM-AS1* in MCF7 and T47D cells. qPCR expression normalized to shControl. Error bars represent the s.e.m. for three biological replicates.

Blot is representative of three independent experiments. ns: $p > 0.01$, comparing to shControl for each condition via Student's t-test. (**c** and **d**) qPCR expression of *DSCAM-AS1* following overexpression of *LacZ* control and *DSCAM-AS1* in (**c**) T47D cells and (**d**) ZR75-1 cells. **: $p < 0.001$, comparing to LacZ overexpression via Student's t-test. (**e**) Invasion assay following overexpression of *LacZ* control and *DSCAM-AS1* in ZR75-1 cells. Error bars represent the s.e.m. for three biological replicates. **: $p < 0.001$, comparing to LacZ overexpression via Student's t-test. (**f**) qPCR expression of *DSCAM-AS1* following overexpression of *LacZ* control and *DSCAM-AS1* in MDA-MB-231 cells. ***: $p < 0.0001$, comparing to LacZ overexpression via Student's t-test. (**g**) Incucyte proliferation assay performed following overexpression of *DSCAM-AS1* in MDA-MB-231 cells. Error bars represent the s.e.m. for three biological replicates. ns: $p > 0.01$ comparing to LacZ overexpression via Student's t-test. (**h**) Invasion assay following overexpression of *LacZ* control and *DSCAM-AS1* in MDA-MB-231 cells. Error bars represent the s.e.m. for three biological replicates. ns: $p > 0.01$, comparing to LacZ overexpression via Student's t-test.

Supplementary Figure 5

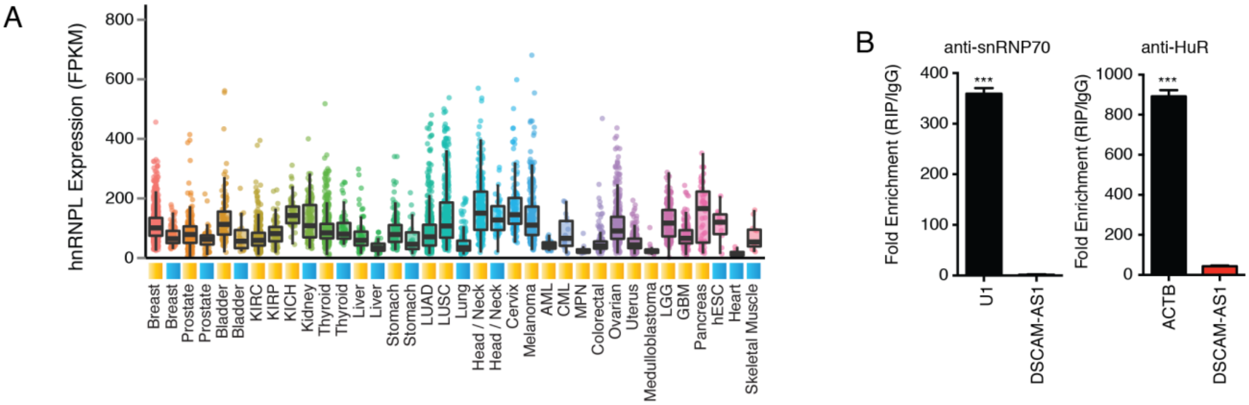

Supplementary Figure 5. HnNRPL binds to and phenocopies *DSCAM-AS1*. (**a**) Expression of hnRNPL in the 6,503 sample MiTranscriptome RNA-seq compendium[13] categorized by the different cancer/tissue types. Each point represents one RNA-seq tissue sample. (**b**) RIP-qPCR for pulldown of snRNPL70 and HuR. *U1* is used as a positive control for snRNP70 pulldown, and *ACTB* is used as a positive control for HuR pulldown. Error bars represent s.e.m. for three biological replicates. ***: $p < 0.0001$, comparing to DSCAM-AS1 fold enrichment via Student's t-test.

Supplementary Figure 6

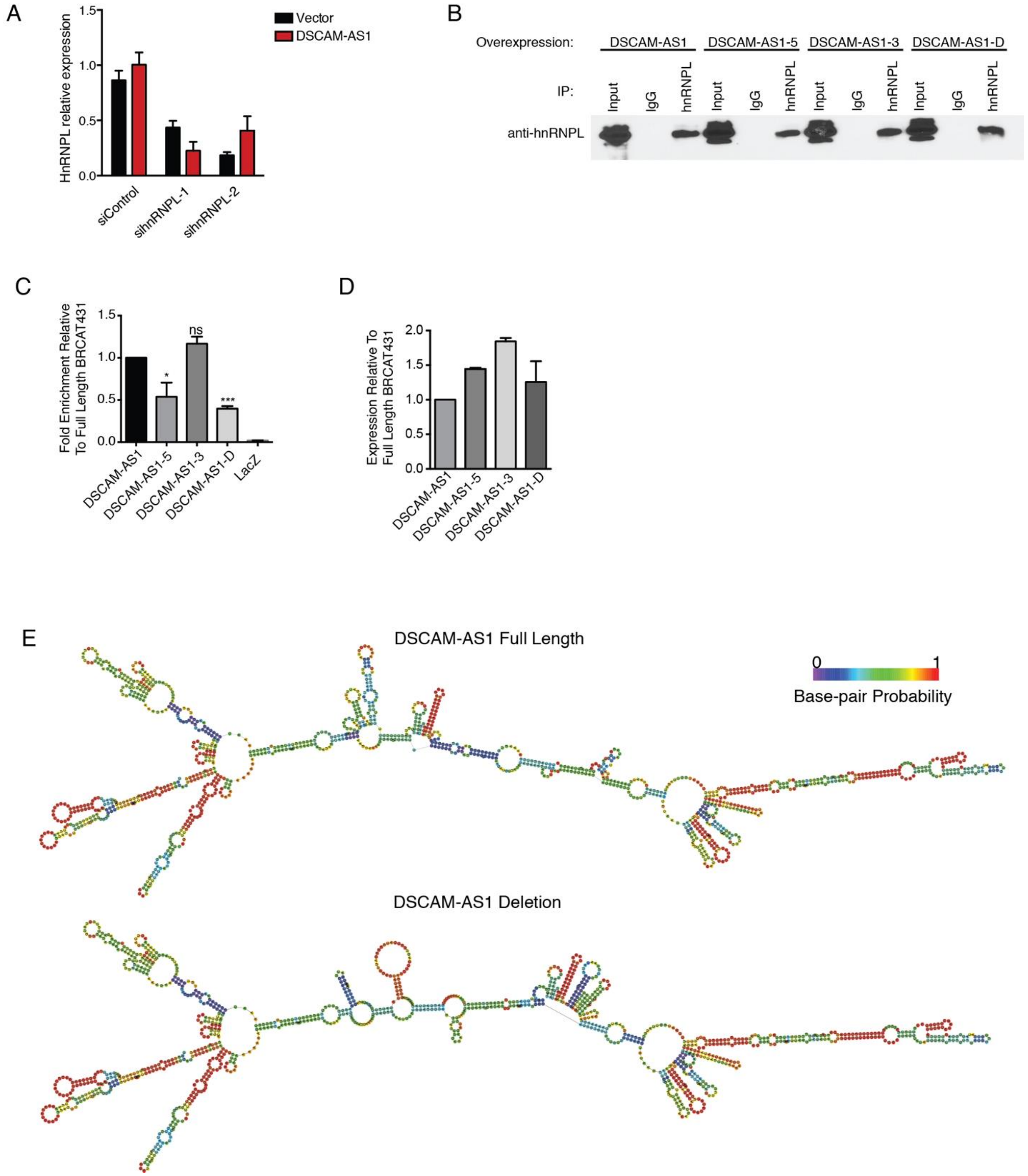

Supplementary Figure 6. Binding of hnRNPL localized to region at 3' end of *DSCAM-AS1*. (**a**) qPCR expression levels of *hnRNPL* following siRNA knockdown of *hnRNPL* in T47D cells overexpressing

DSCAM-AS1 using two independent siRNAs. (**b**) Western blot for hnRNPL following pulldown of hnRNPL in HEK293 cells expressing the wildtype and mutant forms of DSCAM-AS1, confirming hnRNPL expression in these cells. (**c**) qPCR for the wildtype and mutant forms of *DSCAM-AS1* following hnRNPL RIP in HEK293 cells. Expression normalized to fold-enrichment (hnRNPL RIP/ IgG RIP) of full length DSCAM-AS1. *: $p < 0.01$, ***: $p < 0.0001$, ns: $p > 0.01$, comparing to DSCAM-AS1 full length fold enrichment via Student's t-test (**d**) Expression of wildtype and mutant *DSCAM-AS1* by qPCR in HEK293 cells. (**e**) RNA-fold minimum free energy secondary structure prediction for DSCAM-AS1 full length and the DSCAM-AS1-D deletion construct. Color represents base-pair probability.

Supplementary Figure 7

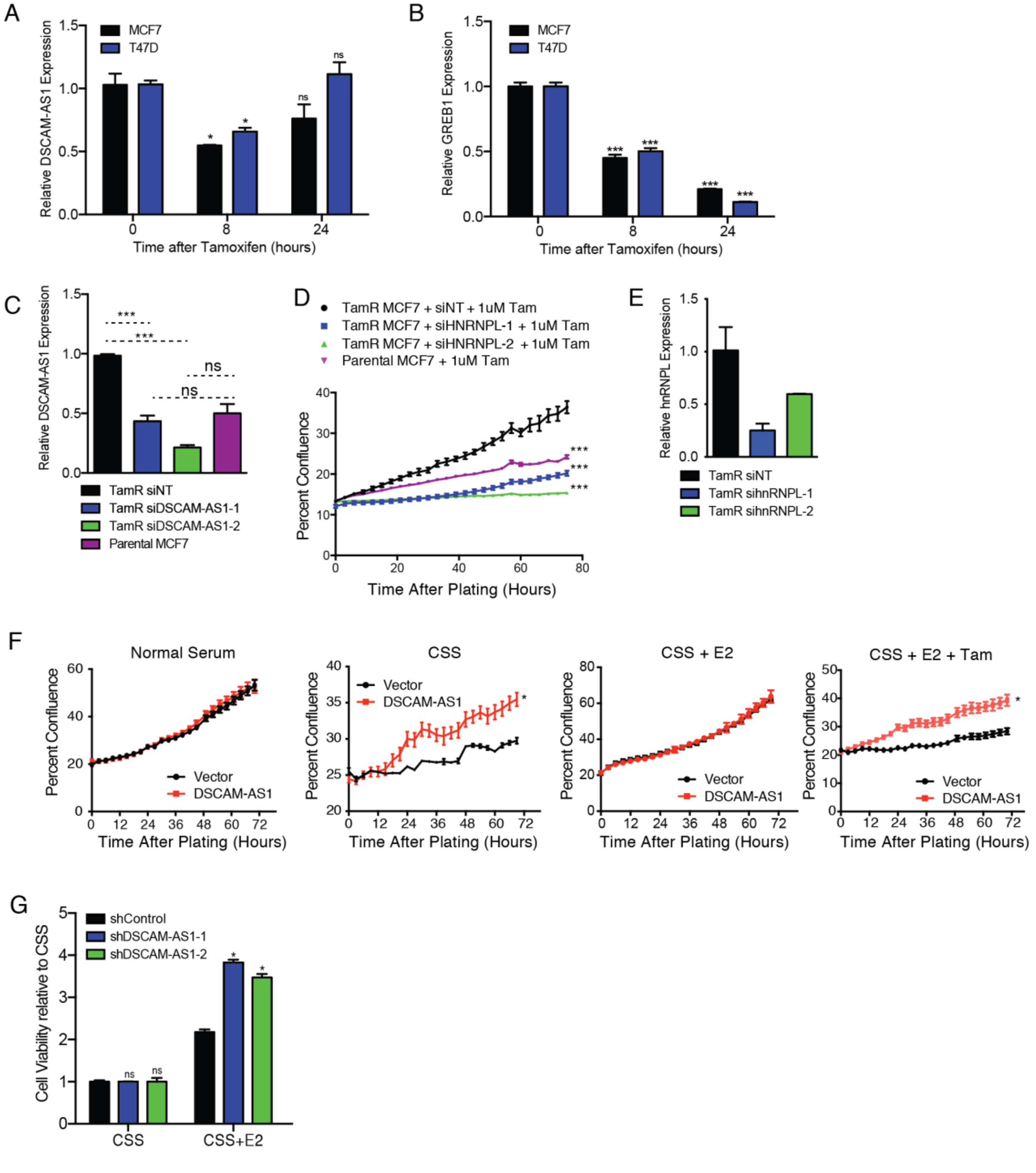

Supplementary Figure 7. *DSCAM-AS1* and hnRNPL mediate estrogen independent growth in breast cancer cells. (**a** and **b**) qPCR expression of (**a**) *DSCAM-AS1* and (**b**) *GREB1* following addition of 1.25 uM tamoxifen in parental MCF7 cells. *: $p < 0.01$, ***: $p < 0.0001$, ns: $p > 0.01$, comparing to 0hr in each condition via Student's t-test (**c**) qPCR expression of *DSCAM-AS1* in TamR MCF7 cells following

siRNA knockdown of *DSCAM-AS1*. Expression plotted relative to non-targeting siRNA control. (**d**) Proliferation assay in parental MCF7 cells and in TamR MCF7 cells following siRNA-mediated knockdown of *hnRNPL* via two independent siRNAs. (**e**) qPCR expression of *hnRNPL* in TamR MCF7 cells following siRNA knockdown of *hnRNPL*. Expression plotted relative to non-targeting siRNA control. (**f**) Proliferation assay comparing T47D cells overexpressing *DSCAM-AS1* versus *LacZ* control in the presence of normal estrogen-containing serum, charcoal stripped serum (CSS), charcoal stripped serum with estrogen (CSS + E2), and charocoal stripped serum with estrogen and 1.25uM tamoxifen (CSS + E2 + Tam). *: $p < 0.01$, ns: $p > 0.01$, comparing to vector overexpression in each condition via Student's t-test. (**g**) Crystal violet cell viability assay following 10 days of culture in charcoal stripped serum (CSS), or charcoal stripped serum with estrogen (CSS + E2) with shRNA knockdown of DSCAM-AS1. Error bars represent the s.e.m. for three biological replicates. *: $p < 0.01$, ns: $p > 0.01$, comparing to shControl in each condition via Student's t-test.

Supplementary Figure 8

Full Blots for Figure 4c

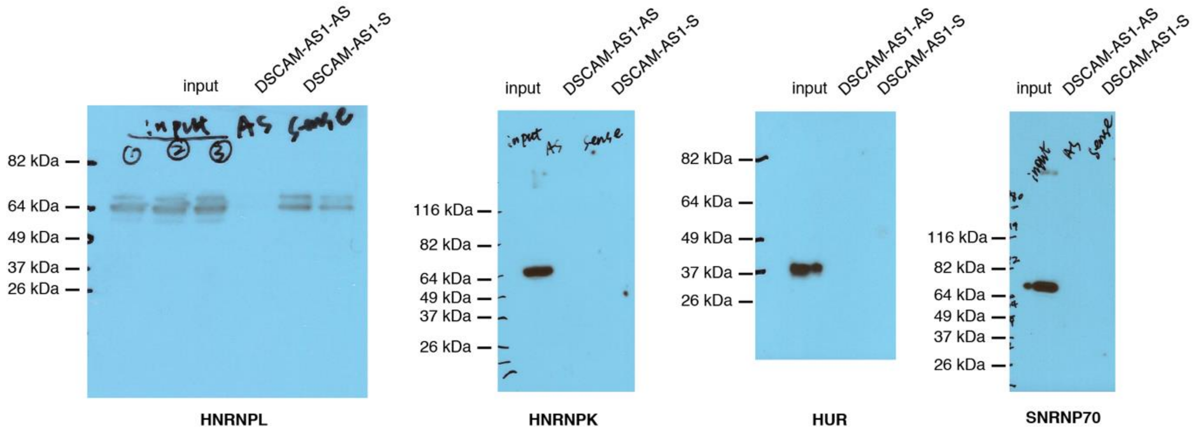

Full Blot for Figure 4g

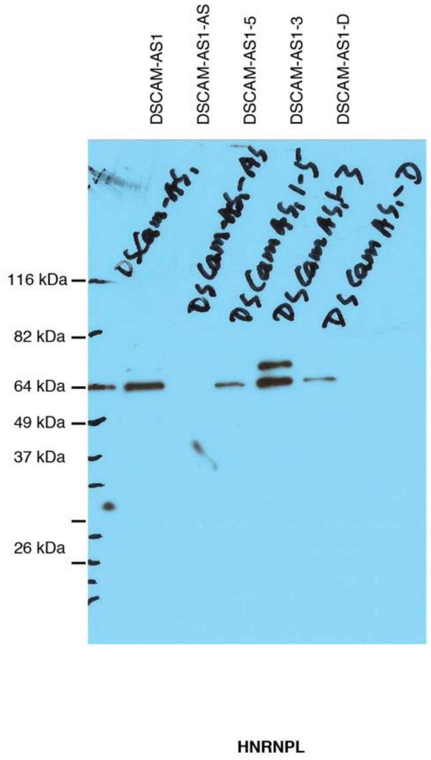

Supplementary Figure 8. Representative full Western blot images presented in Figure 4c,g.
